# Remote Source Document Verification in Two National Clinical Trials Networks: A Pilot Study

**DOI:** 10.1371/journal.pone.0081890

**Published:** 2013-12-05

**Authors:** Meredith Mealer, John Kittelson, B. Taylor Thompson, Arthur P. Wheeler, John C. Magee, Ronald J. Sokol, Marc Moss, Michael G. Kahn

**Affiliations:** 1 Division of Pulmonary Sciences and Critical Care Medicine, Department of Medicine, University of Colorado School of Medicine, Aurora, Colorado, United States of America; 2 Department of Biostatistics and Informatics, Colorado School of Public Health, Aurora, Colorado, United States of America; 3 Colorado Clinical and Translational Sciences Institute, University of Colorado Anschutz Medical Center, Aurora, Colorado, United States of America; 4 Massachusetts General Hospital, Biostatistics Center, Boston, Massachusetts, United States of America; 5 Vanderbilt University Medical Center, School of Medicine, Nashville, Tennessee, United States of America; 6 University of Michigan, Department of Surgery, Ann Arbor, Michigan, United States of America; 7 Department of Pediatrics, University of Colorado School of Medicine, Aurora, Colorado, United States of America; University of New South Wales, Australia

## Abstract

**Objective:**

Barriers to executing large-scale randomized controlled trials include costs, complexity, and regulatory requirements. We hypothesized that source document verification (SDV) via remote electronic monitoring is feasible.

**Methods:**

Five hospitals from two NIH sponsored networks provided remote electronic access to study monitors. We evaluated pre-visit remote SDV compared to traditional on-site SDV using a randomized convenience sample of all study subjects due for a monitoring visit. The number of data values verified and the time to perform remote and on-site SDV was collected.

**Results:**

Thirty-two study subjects were randomized to either remote SDV (N=16) or traditional on-site SDV (N=16). Technical capabilities, remote access policies and regulatory requirements varied widely across sites. In the adult network, only 14 of 2965 data values (0.47%) could not be located remotely. In the traditional on-site SDV arm, 3 of 2608 data values (0.12%) required coordinator help. In the pediatric network, all 198 data values in the remote SDV arm and all 183 data values in the on-site SDV arm were located. Although not statistically significant there was a consistent trend for more time consumed per data value (minutes +/- SD): Adult 0.50 +/- 0.17 min vs. 0.39 +/- 0.10 min (two-tailed t-test p=0.11); Pediatric 0.99 +/- 1.07 min vs. 0.56 +/- 0.61 min (p=0.37) and time per case report form: Adult: 4.60 +/- 1.42 min vs. 3.60 +/- 0.96 min (p=0.10); Pediatric: 11.64 +/- 7.54 min vs. 6.07 +/- 3.18 min (p=0.10) using remote SDV.

**Conclusions:**

Because each site had different policies, requirements, and technologies, a common approach to assimilating monitors into the access management system could not be implemented. Despite substantial technology differences, more than 99% of data values were successfully monitored remotely. This pilot study demonstrates the feasibility of remote monitoring and the need to develop consistent access policies for remote study monitoring.

## Introduction

Large randomized controlled trials (RCT) are the gold standard for evaluating the risk/benefit profile associated with new medical therapies. Numerous publications have described substantial barriers to conducting such trials, including an explosive growth in the cost, complexity, and regulatory requirements[1,2]. Over a four year period, overall clinical trial costs have risen by 70% and the average cost per subject enrolled into Phase III trials increased from $26,000 to over $40,000[3]. Many processes in the conduct of clinical trials are inefficient, thereby prolonging the time for evaluation of therapies and increasing their cost. Recent proposals have focused on either re-engineering how RCTs are performed or replacing them with potentially less robust models such as observational pragmatic trial designs[4–6].

Clinical trial monitoring is a critical process in executing RCTs that is employed by study sponsors to oversee the progress of a clinical trial and to protect human subjects (www.fda.gov). Currently, oversight requires a study monitor to travel to the clinical site[7] to perform a number of tasks designed to ensure the validity and integrity of the clinical trial results regardless of the intervention being tested [8,9] (Table 1). A large component of on-site monitoring is source document verification (SDV), which a) involves the comparison of case report values against the original documentation to verify that reported data are accurate, complete, and verifiable; b) confirms that the trial conduct complies with the protocol and good clinical practice (GCP); and c) ensures that appropriate regulatory requirements have been followed. One study estimated that 46% of a monitor’s on-site time is spent performing SDV[10]. According to a recent literature review, the average data entry/transcription error rate was 976 errors per 10,000 data values, highlighting that data verification is a critical component to ensuring high quality study results[11]. 

**Table 1:** Typical on-site study monitoring tasks.

1. Ensure appropriate communication between the principal investigator (PI) and the sponsor
2. Ensure appropriate communication between the principal investigator (PI) and the sponsor
3. Verify adequate qualifications and resources of the study team
4. Verify storage and accountability of the investigational product
5. Verify proper adherence and conduct of the protocol
6. Verify compliance with the written informed consent document process for subject participation (*)
7. Ensure that the PI and study staff are adequately informed about the trial
8. Verify that the PI in enrolling only eligible subjects (*)
9. Report subject recruitment rate (*)
10. Verify regulatory compliance
11. Determine appropriate reporting of adverse events
12. Communicate protocol deviations and develop an appropriate plan to prevent their recurrence
13. Ensure accuracy and completeness of the case report form (CRF) entries through source document verification (SDV) (*)

From [17] Items noted with '(*)' are amenable to being performed using remote access technologies.

Because of the time intensive nature of study monitoring, it is estimated that approximately 40% of the average costs for all Phase II or Phase III clinical trials are dedicated to SDV[10]. As the number of study sites increases, the cost and logistics of on-site study monitoring trips become burdensome. Many sponsors utilize a fixed unit price budget, setting the total number of hours available to complete all monitoring tasks during an on-site visit. This budgetary constraint may result in less time being spent on other equally important study monitoring responsibilities that require an on-site presence such as study drug accountability, verification of subject eligibility, and compliance with the written informed consent process. 

Secure remote access to electronic health records, directly or via clinician portals, is generally available in most healthcare environments but access has been limited to clinical personnel employed by the institution who are given access to support clinical care. This same technology could provide an alternative approach for many monitoring tasks currently performed during an on-site visit. In particular, SDV, the most time-consuming on-site task, could potentially be done via secure remote access. The logistical convenience and lower costs associated with electronic health records remote access could enable more frequent site monitoring. Reducing the time between site monitoring could enhance the accuracy and timeliness of study data and increase safety to human subjects participating in a clinical trial.

We hypothesized that source document verification via remote monitoring, using existing technologies that support secure off-site electronic health records access for clinical care, was feasible. We conducted a pilot study to better understand the feasibility, accuracy, and efficiency of SDV via secure remote access compared to traditional on-site monitoring techniques across multiple study sites participating in two large NIH-supported multi-institutional clinical trial networks.

## Methods

Two NIH-sponsored clinical trial networks assisted with this study. The ARDS network is funded by the National Heart, Lung, and Blood Institutes of Health to conduct multi-center clinical trials for patients with acute lung injury. We engaged two clinical ARDS network centers at the University of Colorado Anschutz Medical Center and Vanderbilt University. These two clinical centers represented 4 study sites (University of Colorado Hospital, Denver Health Medical Center, St. Anthony’s Central, and Vanderbilt Medical Center). The Childhood Liver Disease Research and Education Network (ChiLDREN) is funded by the National Institute of Diabetes and Digestive and Kidney Diseases. This network conducts multi-center trials that focus exclusively on rare pediatric liver diseases. Children’s Hospital Colorado is a ChiLDREN network site and the Administrative Core. The ARDS and ChiLDREN networks have clinical coordinating centers responsible for monitoring the conduct of the network’s clinical trials. 

The ARDS network’s monitoring plan includes a technical review (chart review) and a scientific review (organizational peer review) every two years. The technical review is performed by a research nurse (study monitor) from the clinical coordinating center, focuses on SDV, regulatory documents and compliance with GCP. A random sample of approximately 10% of enrolled subjects is reviewed to confirm eligibility and proper informed consent, and validate that all adverse events are reported. All data values related to primary and secondary outcome variables are confirmed against the source documents. The study intervention is reviewed including drug administration or performance of study procedures. Finally, a random sample of on-study data is reviewed. The ChiLDREN network has a different set of policies and procedures for study monitoring. 

Within the ChiLDREN Network, a project manager from the Data Coordinating Center is responsible for clinical site monitoring. Clinical site visits are conducted every one to two years depending on site enrollment and the specifics of the studies being conducted by the Network. During this time period, ChiLDREN was conducting several prospective observational longitudinal cohort studies as well as a randomized double blind placebo controlled interventional trial of corticosteroids for infants with biliary atresia. Monitoring site visits are typically two to three days in duration, during which time the project manager focuses on review of regulatory documents related to all studies. Source data confirming subject eligibility is inspected. Monitoring the interventional trial is a primary focus, and there was 100% source verification of data directly related to primary and secondary endpoints. In addition, drug reconciliation logs are reviewed with the site research pharmacy. Time permitting, a random sample of key endpoints related to the observational studies is performed. 

**Figure 1:**
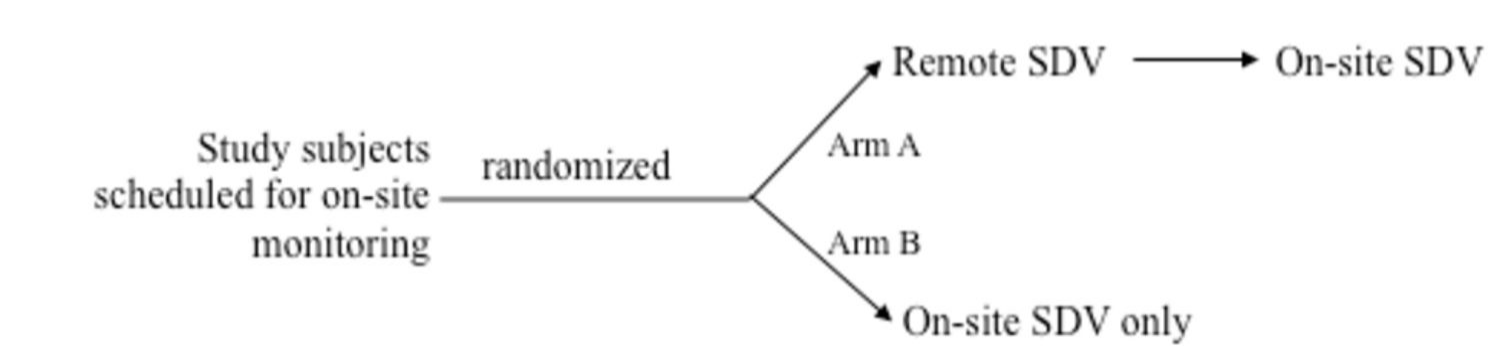
Pilot study design. Nine adult and seven pediatric subjects scheduled for routine monitoring were randomly assigned to Arm A. The same number of study subjects in each network was randomly assigned to Arm B.

We evaluated the feasibility, accuracy, and efficiency of pre-visit remote SDV by study monitors followed by on-site verification compared to traditional on-site SDV by study monitors by randomizing study subjects participating in one ARDS and one ChiLDREN clinical trial who were scheduled for an upcoming on-site monitoring visit (Figure 1). 

Pre-study planning included obtaining remote access for the study monitor to all required systems, which required administrative/regulatory/security approvals and technical reviews to document appropriate security and auditing capabilities. Data collection instruments for capturing study variables were developed and pilot tested using the REDCap data management system[12]. Standard procedures were developed for when to stop searching for a data value, how to account for work breaks and other interruptions, and when to call for additional help from local site managers. 

For this pilot study, we chose all of the Denver hospitals participating in the ARDS or ChiLDREN Network plus an ARDS Network site in Tennessee (n=5), that had an electronic medical record. A convenience sample, consisting of all study subjects who were due for an upcoming monitoring visit that included source document verification, was randomized into two equal arms and stratified at each individual hospital (). Subjects were assigned to having remote SDV performed 2-4 weeks prior to a scheduled on-site visit (Arm A) or having no pre-visit remote SDV performed (Arm B). Arm B had traditional on-site SDV and study monitoring performed whereas Arm A had on-site SDV performed only for data values that could not be verified via remote monitoring. For each research network, the same monitor performed both remote and local monitoring. 

Remote access for the study monitor was set up by technical staff at the five study locations to allow monitors to access electronic records securely over the Internet. Four of the five sites had the ability to limit the monitor's access to only study subjects assigned to the remote arm. Monitors were trained on the specific remote access procedures for each site using web-based remote desktop sharing and a mock study subject who was not scheduled for monitoring. Remote SDV validated the data elements captured on case report forms submitted to the coordinating center using the same data verification protocols that were used during on-site visits. Remote monitors had telephone access to the same local coordinators that were available during on-site monitoring visits.

To assess the ability of a monitor to verify the data value that was recorded on the study case report form, six possible verification outcome states were defined (Table 2). In particular, Outcome #5 (“Found-match after coordinator query”) represents the case where remote access was insufficient to find a data value what was found during the subsequent on-site inspection. Data values not scheduled for verification were assigned the “Not Monitored” outcome. After on-site verification, all data values were assigned only one outcome state. 

**Table 2:** Outcome definitions for source document verification.

Outcome	Definition
1. Found-match	Data value recorded on the case report form matched the data value in the source document.
2. Found-different	Data value recorded on the case report form was different (did not match) the data value in the source document.
3. Missing	Data value recorded on the case report form could not be found in the source document
4. Unknown	No data on the case report form or in the source document related to a data value that was supposed to be collected
5. Found-match after coordinator query	Data value entered in the case report form could not be verified by the study monitor alone. After the study monitor spoke with the study coordinator, the data value was found and matched to the source document
6. Not monitored	Data value was not verified by the study monitor.

Using a time diary that recorded start/stop time intervals, the total time required for the study monitor to verify a case report form was captured. Recorded intervals included time estimates for finding charts, making copies, and recording data. Separate time estimates were recorded if a study coordinator participated in monitoring activities other than SDV. The same data capture definitions and procedures and were used for remote and on-site SDV.

### Statistical design and analysis

The study sites (4 adult hospitals and Children’s hospital) were pre-selected as described above. Of the 94 adult and 12 children who were currently participating in ARDS or ChiLDREN studies, the study was planned to evaluate SDV for 4 subjects in each of the 5 sites (20 total; 10 per arm) but ultimately evaluated SDV for 32 subjects (16 randomized to on-site and 16 to remote SDV). The primary comparison was based on a 2-sample (unequal variance) level α=0.05 (2-sided) t-test with a sample size that provides over 80% power for a difference between groups of 45 minutes (0.75 hours) based on a preliminary time data from 8 cases, which showed an average of 4.3 hours per CRF with a standard deviation of 0.45 hours. The accuracy and completeness of remote SDV versus on-site monitoring was determined by analyzing the number of data values assigned to Outcomes #1-#4 (Table 2) compared to all data values other than those assigned to the “Not Monitored” outcome (Outcome #6). Efficiency was measured by analyzing the amount of time it took to complete the SDV tasks both by individual data item and by CRF form. 

The Colorado Multiple Institutional Review Board approved this study and determined that the study was non-human subject research. 

## Results

The five study sites had significantly different health information technology infrastructures and applications, resulting in different approaches to enabling remote access and remote data monitoring (Table 3). None of the five participating hospitals used the same electronic health record, clinical data repository, web-based access technologies, or authentication and auditing tools. Only one facility used only commercial products. The other organizations created custom solutions for at least one function for remote access.

**Table 3:** Technologies, data domains, and remote access policies at five study sites.

**Study Site **	**Access**	**Data Source**	**Data Domains**	**Access Policies**
A	Home grown web portal	Home grown data repository	Clinical laboratory results, Diagnostic reports (e.g. radiology results) Added clinical documentation, flow sheets and bedside instrument (ventilator) settings	Security agreements, vendor agreement, BAA
B	Vendor A web application #1 (CDR)	Vendor C CDR	Structured clinical data, Diagnoses, Laboratory results, Radiology/pathology reports, Physician notes	Security agreements, BAA
B	Vendor B web application #2 (clinical documentation)	Vendor C CDR	Structured clinical data, diagnoses, laboratory results, radiology/pathology reports, physician notes	Security agreements, BAA
C	Vendor clinical portal	Vendor D EHR	All clinical domains (comprehensive EHR)	Security agreements
D	Secure screen sharing	Home grown EHR	All clinical domains (comprehensive EHR)	Security agreements, BAA
E	Vendor E remote HIM module	Vendor E EHR	All clinical domains (comprehensive EHR)	Security agreements, vendor agreements, BAA

BAA = business associates agreement; CDR = clinical data repository; EHR = electronic health record; HIM = health information management

Eighteen study subjects were randomized between Arm A (N=9) and Arm B (N=9) for the ARDS Network. There were 68 possible case report forms but each study subject had only a subset of case report forms monitored. The specific case report forms and specific data values within case report forms verified were based on which forms had been monitored in previous visits and patient status. Thirteen case report forms were not examined in any patient. Fourteen study subjects (N(Arm A) = 7; N(Arm B)=7) were randomized for the pediatric ChilDREN network. There were 2 case report forms. As with the adult network, the specific data values verified in each case report form varied by patient. These case report forms are representative of longitudinal observational (non-interventional) multi-institutional studies.

Table 4 summarizes the findings for the 5,954 data values verified across all five hospitals. Of the 2,965 data values in the remote SDV arm for the adult network, only 14 data values (0.47%; exact 95% confidence interval: 0.03% to 0.79%) could not be located remotely but were located during the on-site visit. Three data values (0.13%; exact 95% confidence interval: 0.03% to 0.37%) in the on-site only group also required help from the on-site study coordinator. In the pediatric network, all 198 data values in the remote source documentation arm were located remotely. All 183 data values in the on-site only arm were also found.

**Table 4:** Number of data values verified from Remote + On-site versus On-site-Only source document verification.

		Found-match	Found-different	Found-match after coordinator query
ARDS Network (adult)	Remote+On-site (N=9)	2630	321	14
ARDS Network (adult)	On-site only (N=9)	2343	262	3
ChiLDREN Network (pediatric)	Remote+On-site (N=7)	185	13	0
ChiLDREN Network (pediatric)	On-site only (N=7)	182	1	0

Key: Found-match: value found via remote access; value is the same as value in CRF. Found-different: value found via remote access; value is different from value in CRF. Found-match after coordinator query: value not found via remote access; value found with help from local coordinator.

In the ARDS network, the specific CRFs and data elements verified varied by patient. In the ChilDREN network, the specific data elements verified in 2 CRFs varied by patient.

Table 5 summarizes the results of the times for performing source document verification. The results show a consistent trend for more time consumed per data item and time per case report form for both the adult and pediatric networks using remote+on-site SDV, although the observed differences were neither large (less than 0.5 minutes per data item) nor statistically significant. 

**Table 5:** Time to complete Remote+On-site versus On-site Only source document verification.

		Remote + on-site (minutes ± sd)	On-site only (minutes± sd)	Confidence Intervals	p-value
ARDS Network (adult)	Time per item	0.50 ± 0.17	0.39 ± 0.10	-0.25-0.03	0.11
ARDS Network (adult)	Time per CRF page	4.60 ± 1.42	3.60 ± 0.96	-2.22-0.23	0.10
ChiLDREN Network (pediatric)	Time per item	0.99 ± 1.07	0.56 ± 0.61	-1.48-0.61	0.38
ChiLDREN Network (pediatric)	Time per CRF page	11.64 ± 7.54	6.07 ± 3.18	-12.70-1.56	0.10

## Discussion

The goal of clinical trial monitoring is to avoid errors that may compromise patient safety and study results[13]. On-site monitoring is expensive; remote monitoring has the potential to significantly reduce the resources required to meet basic monitoring practices. Remote source document verification can enable more frequent monitoring of trial integrity and could improve safety concerns by detecting errors earlier than would be possible with more limited on-site monitoring. For example, with remote SDV, the study sponsor has the ability to more frequently evaluate patient safety events, identify early safety signals, and check protocol compliance issues such as inclusion/exclusion compliance for subject recruitment, correct dosing of study medications through drug accountability monitoring and appropriate timing and response to safety laboratory blood testing[14]. This is in direct contrast to traditional on-site monitoring efforts that employ varying intervals between monitoring visits. While this study only addressed remote monitoring for SDV, the opportunity presents itself to use remote access for additional features of study monitoring, including study drug accountability, consent documentation, and basic regulatory documentation, as well as for training.

An informal post-study interview of the study monitors and site coordinators involved in the pilot study revealed a high level of satisfaction with the remote monitoring process and a strong desire to expand its use at other sites in both national networks. Neither study monitor had difficulty with using different electronic access methods and data review applications.

There was a consistent increase in overall time for remote source document verification versus traditional on-site monitoring; although differences were not statistically significant, the magnitude of per-item differences was very small (less than 30 seconds). The practical impact of time differences on a given trial would also have to consider travel time for study monitors, which was not quantified in this study, and could vary substantially across networks or trials. The major reason noted for the additional overall time was delayed response time between the study monitor and site coordinator for responding to questions. During on-site visits, the monitor and site coordinator meet at pre-scheduled times to review questions that have accumulated during the review. Remote monitoring allows more fluid workflows but also requires more coordination to ensure rapid turnaround times in response to monitor questions. Implementing different workflows/Standard Operating Procedures, such as pre-scheduling times to review questions over the telephone or incorporating the use of chat/instant messaging for more interactive communications, could potentially ameliorate delays in responses. Also, it is reasonable to believe that repeated exposure to remote monitoring and accessing the electronic health record applications would translate into improved efficiency, as would use of a single electronic health record.

The sites in this study may not generalize to other institutions; however, each site had significantly different health information technology infrastructures and applications with markedly differing user interfaces and functionality. The diversity of applications and interfaces was an important aspect of this study as it emulated the diversity in health information technologies across research locations. One additional potential advantage to remote source document verification is the ability to expand study sites to include more difficult-to-reach smaller practices. As the implementation of electronic health records accelerates in the United States, more physician practices will have the ability to support remote access. If additional study monitoring tasks could be supported via remote access, these sites, which previously were too expensive to include in research studies, could become a new source of study subjects. Our study is also limited by the non-blinded randomization method chosen. However, if each case report form was exposed to both remote and on-site monitoring, there is a potential for bias in time to complete the monitoring tasks due to familiarity with the record.

Although not the focus of our study, shows that 583 data values (10.5%) of the 5,573 data values that were found in either arm in the ARDS network had values different in the source document compared to the case report form. For the ChiLDREN network, there were 14 data values (3.7%) across 381 found variables that were different in the source document compared to the case report form. These results are consistent with findings from traditional on-site source document verification, highlighting the importance and value of this component of clinical trial monitoring[11,13,15,16].

## Conclusions

The results of this pilot study suggest that source document verification via remote monitoring is feasible. In the adult network, 99.5 percent of all data values were found remotely and 100% of the children’s network data values were verified remotely. Remote SDV was feasible despite marked differences in remote access and remote chart review policies and technologies. While this study only addressed remote monitoring for SDV purposes, the opportunity exists to encompass additional features of study monitoring, such as study drug accountability, consent documentation, and basic regulatory documentation, and the potential to reduce the cost of this important aspect of clinical trial monitoring. 

## References

[1] Spaar A, Frey M, Turk A, Karrer W, Puhan MA (2009) Recruitment barriers in a randomized controlled trial from the physicians’ perspective: a postal survey. BMC Med Res Methodol 9: 14. doi:10.1186/1471-2288-9-14. PubMed: 19254374. 19254374 PMC2653070

[2] Duley L, Antman K, Arena J, Avezum A, Blumenthal M et al. (2008) Specific barriers to the conduct of randomized trials. Clin Trials 5: 40–48. doi:10.1177/1740774507087704. PubMed: 18283079. 18283079

[3] Edge Cutting. Information (2013) Per-Patient; Clinical Trial Costs Rise 70% in Three Years. Available: http://www.cuttingedgeinfo.com/2011/per-patient-clinical-trial-costs/ . Accessed 15 May 2013

[4] Concato J, Shah N, Horwitz RI (2000) Randomized, controlled trials, observational studies, and the hierarchy of research designs. N Engl J Med 342: 1887–1892. doi:10.1056/NEJM200006223422507. PubMed: 10861325. 10861325 PMC1557642

[5] Relton C, Torgerson D, O’Cathain A, Nicholl J (2010) Rethinking pragmatic randomised controlled trials: introducing the “cohort multiple randomised controlled trial”. Design - BMJ 340: c1066. 20304934 10.1136/bmj.c1066

[6] Thorpe KE, Zwarenstein M, Oxman AD, Treweek S, Furberg CD et al. (2009) A pragmatic-explanatory continuum indicator summary (PRECIS): a tool to help trial designers. CMAJ Can Med Assoc J J Assoc Medicale Can 180: E47–E57. doi:10.1503/cmaj.090523. PMC2679824 19372436

[7] Spilker B (1991) Guide to Clinical Trials. New York, NY: Raven Press.

[8] Davis JR, Institute of Medicine (U.S.). Roundtable on Research and Development of Drugs Biologics and Medical Devices. (1999) Assuring data quality and validity in clinical trials for regulatory decision making : workshop report. Washington, DC: National Academy Press . xii, 76 p. p. Available: http://books.nap.edu/catalog/9623.html. 25077226

[9] Williams GW (2006) The other side of clinical trial monitoring; assuring data quality and procedural adherence. Clin Trials 3: 530–537. doi:10.1177/1740774506073104. PubMed: 17170037. 17170037

[10] Breslauer C (2006) Could source document verification become a risk in a fixed-unit price environment? Monitor: 43–47.

[11] Nahm ML, Pieper CF, Cunningham MM (2008) Quantifying data quality for clinical trials using electronic data capture. PLOS ONE 3: e3049. doi:10.1371/journal.pone.0003049. PubMed: 18725958. 18725958 PMC2516178

[12] Harris PA, Taylor R, Thielke R, Payne J, Gonzalez N et al. (2009) Research electronic data capture (REDCap)--a metadata-driven methodology and workflow process for providing translational research informatics support. J Biomed Inform 42: 377–381. doi:10.1016/j.jbi.2008.08.010. PubMed: 18929686. 18929686 PMC2700030

[13] Baigent C, Harrell FE, Buyse M, Emberson JR, Altman DG (2008) Ensuring trial validity by data quality assurance and diversification of monitoring methods. Clin Trials 5: 49–55. doi:10.1177/1740774507087554. PubMed: 18283080. 18283080

[14] Stafford PB, Garrett A (2011) Using Real-time Data to Drive Better Decisions, Faster. Drug Inf J 45: 495–502. doi:10.1177/009286151104500410.

[15] Horbar JD, Leahy KA (1995) An assessment of data quality in the Vermont-Oxford Trials Network database. Control Clin Trials 16: 51–61. doi:10.1016/0197-2456(94)00019-Y. PubMed: 7743789. 7743789

[16] Vantongelen K, Rotmensz N, van der Schueren E (1989) Quality control of validity of data collected in clinical trials. EORTC Study Group on Data Management (SGDM). Eur J Cancer Clin Oncol 25: 1241–1247. doi:10.1016/0277-5379(89)90421-5. PubMed: 2767111. 2767111

[17] ICH Harmonised Tripartite Guideline (1996) Guildeline for Good Clinical Practice E6 (R1). Available: http://www.ich.org/fileadmin/Public_Web_Site/ICH_Products/Guidelines/Efficacy/E6_R1/Step4/E6_R1__Guideline.pdf . Accessed 1 June 2013

